# Editorial: Post COVID-19: the nucleoside-modified messenger RNA (modRNA) platform

**DOI:** 10.3389/fmed.2023.1324610

**Published:** 2024-01-04

**Authors:** Luca Roncati, Qun Treen Huo

**Affiliations:** ^1^Department of Surgery, Medicine, Dentistry and Morphological Sciences with Interest in Transplantation, Oncology and Regenerative Medicine, University of Modena and Reggio Emilia, Modena, Italy; ^2^Department of Laboratory Medicine and Anatomical Pathology, Institute of Pathology, University Hospital of Modena – Polyclinic, Modena, Italy; ^3^Department of Chemistry, University of Central Florida, Orlando, FL, United States; ^4^NanoScience Technology Center, University of Central Florida, Orlando, FL, United States

**Keywords:** Coronavirus Disease 2019 (COVID-19) vaccine, messenger RNA (mRNA), nucleoside-modified mRNA (modRNA), nanoparticles, dendritic cells, personalized oncology, phenylketonuria, reproductive biology

## Perspective

The year 2020 marked a turning point in medicine, not only because it was the year of the Coronavirus Disease 2019 (COVID-19) pandemic outbreak, but also because in the same year, and for the first time in history, the Western drug regulatory agencies authorized the emergency use of nucleoside-modified mRNA (modRNA), embedded in lipid nanoparticles as COVID-19 vaccines, hitherto never approved for ethical reasons (1). About 30 years had passed since Malone, Felgner, and Verma of the Salk institute in San Diego succeeded in the feat of transfecting *Photinus pyrais* luciferase mRNA into mouse cells by exploiting lipofectin, an innovative liposome for the era (2). The researchers also noted that the translation of this mRNA could be affected by minor structural changes of the transcripts, paving the way for nucleoside modification (2). At the beginning of the 90s, liposome-incorporated mRNA encoding a viral antigen was proven to induce specific cytotoxic T lymphocytes in recipient mice (3); the same technique was then applied to mice to elicit both cellular and humoral responses against a viral or tumor antigen. The first human clinical trial using autologous dendritic cells transfected with mRNA encoding tumor antigen dates back to 2001–2002 (4); 4 years later, nucleoside modification was shown to be an effective biotechnology in avoiding the hyperactivation of the innate immune system by Toll-like receptors (5). The first human clinical trial against an infectious agent (*Rabies lyssavirus*) began in 2013 (6); over the next few years, clinical trials of mRNA vaccines for other viruses were started, among which Zika, Chikungunya, HIV, Influenza and Ebola (7–11). It's news this year that Karikó and Weissman have been awarded the Nobel Prize in Physiology or Medicine for their discoveries concerning nucleoside base modifications that enabled the development of effective mRNA vaccines against COVID-19 (12).

## Article summary

In this context of great medical relevance, *Frontiers in Medicine* has kept faith to its mission of supporting the translation of scientific advances into new therapies and diagnostic tools that will improve patient care, and its focus has been on exploring the current and potential fields of modRNA application in an interdisciplinary approach, such as vaccinology, cancer therapy, rare diseases, genetically determined illnesses, and enzyme-replacement tools; this Research Topic has been a first step to achieve all the set goals through four new papers (two original research and two review articles).

Jonny et al. from Indonesia have reported the final analysis after 1-year follow-up regarding the safety and efficacy in phase I and phase II clinical trials of personalized vaccines made up from autologous monocyte-derived dendritic cells incubated with the spike protein of the etiological agent of COVID-19, the Severe Acute Respiratory Syndrome Coronavirus 2 (SARS-CoV-2). A total of 28 subjects in the phase I clinical trial were randomly assigned to nine groups based on antigen and granulocyte-macrophage colony stimulating factor dosage. In the phase II clinical trial, 145 subjects were randomly grouped into three groups based on antigen dosage. During the follow-up period, no subjects in phase I experienced moderate-severe COVID-19; meanwhile, about 4% of subjects in phase II had moderate-severe COVID-19. Therefore, after 1-year follow-up, the vaccine has proven safe and effective for preventing COVID-19 (Jonny et al.).

Cacicedo et al. from Germany have tested a novel mRNA-based approach in phenylketonuric (PKU) mice showing a fast reduction in the accumulation of phenylalanine in serum, liver and brain, the organs most affected by the disease. Repeated injections of lipid nanoparticles-formulated mouse phenylalanine hydroxylase mRNA were able to rescue PKU mice from the disease phenotype for a long period of time. Therefore, a mRNA-based approach could significantly improve the quality of life in PKU patients of all ages by replacing standard-of-care treatments in the near future (Cacicedo et al.).

Ladak et al. from Canada have provided an informative update of mRNA vaccines against viruses and cancer. Highly flexible, scalable and cost-effective, mRNA therapy is a compelling vaccine platform against viruses; likewise, mRNA vaccines show similar promise against cancer as a platform capable of encoding multiple antigens for a wide range of cancers, including patient-specific ones, as a new form of personalized oncology (Ladak et al.).

Bafleh et al. from United Arab Emirates have describe key areas where mRNA-based platforms have potential clinical applications, specifically with relation to oocyte and embryo delivery of mRNA to combat infertility in humans, a pioneering approach to exploit RNA therapeutics within reproductive biology (Bafleh et al.).

## Future directions

The modRNA platform represents an ongoing milestone and paradigm shift in modern pharmacology: no longer administering a protein from the outside, but providing the organism with the blueprint to synthesize the same protein from the inside. Thanks to this extraordinary platform, it is even possible to introduce heterologous modRNA into the cytoplasm of cells, bypassing transcription, inducing them to assemble proteins that they do not produce; moreover, by selecting suitable untranslated regions (UTRs) during the synthesis of a modRNA, the amount of the produced protein can be optimized (13). Important future directions of research will therefore concern vaccines based on structural peptides of emerging pathogens, antibodies against tumor antigens of the affected patient, regenerative medicine (including the regeneration of damaged cardiac muscle tissue), a broad spectrum of rare diseases caused by enzyme deficiency, high-performance systems of packaging and nano-delivery of the modRNA, non-standard nucleosides or synthetic analogs to be exploited for mRNA modifications in addition to pseudouridine or N1-methyl-pseudouridine (14, 15), and pharmacovigilance studies (16–21). This revolutionary platform, in fact, should not be exempt from continuous safety and efficacy controls as for any type of drug, always in the interest of patients' health.

## Author contributions

LR: Conceptualization, Data curation, Formal analysis, Methodology, Project administration, Resources, Software, Supervision, Validation, Visualization, Writing—original draft, Writing—review & editing. QH: Project administration, Software, Supervision, Validation, Visualization, Writing—review & editing.
